# pH-Responsive carriers for oral drug delivery: challenges and opportunities of current platforms

**DOI:** 10.1080/10717544.2017.1279238

**Published:** 2017-02-14

**Authors:** Lin Liu, WenDong Yao, YueFeng Rao, XiaoYang Lu, JianQing Gao

**Affiliations:** 1 The First Affiliated Hospital, College of Medicine, Zhejiang University, Hangzhou, PR China,; 2 Institute of Pharmaceutics, College of Pharmaceutical Sciences, Zhejiang University, Hangzhou, PR China, and; 3 College of Pharmaceutical Sciences, Zhejiang Chinese Medical University, Hangzhou, PR China

**Keywords:** pH-Responsive, oral delivery, controlled release, bioavailability, drug delivery

## Abstract

Oral administration is a desirable alternative of parenteral administration due to the convenience and increased compliance to patients, especially for chronic diseases that require frequent administration. The oral drug delivery is a dynamic research field despite the numerous challenges limiting their effective delivery, such as enzyme degradation, hydrolysis and low permeability of intestinal epithelium in the gastrointestinal (GI) tract. pH-Responsive carriers offer excellent potential as oral therapeutic systems due to enhancing the stability of drug delivery in stomach and achieving controlled release in intestines. This review provides a wide perspective on current status of pH-responsive oral drug delivery systems prepared mainly with organic polymers or inorganic materials, including the strategies used to overcome GI barriers, the challenges in their development and future prospects, with focus on technology trends to improve the bioavailability of orally delivered drugs, the mechanisms of drug release from pH-responsive oral formulations, and their application for drug delivery, such as protein and peptide therapeutics, vaccination, inflammatory bowel disease (IBD) and bacterial infections.

## Introduction

Drug administration by oral route is the most ideal choice owing to its simplicity, convenience, minimal pain and suitability (Xu et al., 2013), especially for chronic therapy. It is expected to solve the noncompliance-related problems associated with injections of protein and peptide molecules, improve the bioavailability of poorly water-soluble drugs, and reduce drug-related adverse effects of chemotherapy because of the favorable pharmacokinetics (Pfeiffer et al., 2006). In addition, oral formulations have unique advantages for both physicians and industry, such as flexible dosing schedules, less demands on staff, reduced costs through less hospital or clinic visits, and less expensive production costs, which is especially attractive for pharmaceutical industry (Findlay et al., 2008; De Portu et al., 2010).

However, orally delivered drugs are exposed to a very harmful environment that variations occur in the process of pharmaceutical absorption. First, drugs, especially peptide and protein, may be degraded by a variety of digestive enzymes present in the stomach and small intestine (Sood & Panchagnula, 2001; Goldberg & Gomez-Orellana, 2003). Second, the value of pH in the gastrointestinal (GI) tract is obviously different which varies from highly acidic in the stomach (pH 1–3) to neutral or slightly alkaline in the duodenum (pH 6) and along the jejunum and ileum (pH 6–7.5) (Felber et al., 2012; Xu et al., 2013). Exposure to these pH values can result in hydrolysis, oxidation or deamidation of protein drugs, leading to deactivation (Sood & Panchagnula, 2001). Finally, the intestinal epithelium is the main barrier for the absorption of hydrophilic macromolecules such as peptide, proteins, nucleic acids, and polysaccharides due to their hydrophilicity and high molecular weight, which makes it difficult for them to diffuse across the lipid bilayer cell membranes (Ng et al., 2011; Xie et al., 2011). Conventionally, many drugs, especially therapeutic proteins, are administered subcutaneously, intramuscularly or intravenously since oral administration may cause low bioavailability in the GI tract (Choonara et al., 2014). Accordingly, it has become a challenge to achieve consistent and adequate bioavailability levels for administering orally.

Of varied methods for overcoming the barriers, pH-triggered release mechanisms are extensively used in oral administration. The pH-responsive carriers for oral drug delivery have been proven to enhance the stability of drug delivery in stomach and achieve controlled release in intestines. Our laboratory largely focuses on pH-responsive polymeric systems for oral delivery of drugs, and has successfully developed a pH-responsive and colon-specific capsule which is potential to be used as a reliable carrier for colon-specific drug delivery (Han et al., 2009). In this review, we deal with the possibilities being explored in the pH-responsive oral drug delivery systems prepared from organic polymers or inorganic materials, the challenges in their development and future prospects, with focus on technology trends to improve the bioavailability of orally delivered drugs, the mechanisms of drug release from pH-responsive oral formulations, and their application for drug delivery, such as protein and peptide therapeutics, vaccination, inflammatory bowel disease (IBD) and bacterial infections.

## Formulation approaches for pH-responsive oral delivery systems

### Hydrogels

Hydrogels possess a diversity of tunable features of the bulk structure that can be tailored for a specific therapeutic. Crosslinked hydrogel networks enable to protect drugs from hostile environment, such as low pH and enzymes in the stomach (Qiu & Park, 2001). Density of a crosslinking agent and chemical structure determines the mesh size (ξ), and can be optimized for loading and controlled diffusion of water soluble drugs in or out of the network (Peppas et al., 2000). Incorporation of hydrophilic groups in crosslinking agent can cause higher degree of swelling compared to those containing hydrophobic groups that collapse in water, thus reducing hydrogel swelling.

Drugs release from pH-responsive hydrogels after the materials swelling at specific pH (Tan et al., 2007). Mesh size of the swollen network affects the physical properties of the hydrogel, such as degradation, diffusion of captured molecules, and mechanical strength (Peppas et al., 2000). Mesh size of hydrogels in the swollen state as reported typically ranges from 5 to 100 nm and can be optimized for sustained release of macromolecules based on their hydrodynamic radii (Sharpe et al., 2014). Tan and Tam (2007) found that the changes of particles size depended on the pH of dissolution medium. It swelled when pH was 7.4 and 8, while de-swelling at the pH of 5 and 6.

There are two basic strategies for imparting pH-responsive behavior: i) ionizable groups with solubility and/or conformational changes in response to environmental pH; and ii) acid sensitive bonds that cleave to release molecules anchored into the backbone (Mura et al., 2013). The pH-responsive hydrogels can be classified as anionic or cationic. Anionic hydrogels are ionized, and thus swollen, at a pH above the pKa of the polymer network (Ranjha et al., 2010). Intestinal drug delivery systems protect drugs from gastric degradation and denaturation at low pH and release drugs in specific locations, such as the upper small intestine and colon, further in the GI tract, by taking advantage of pH-responsive anionic hydrogels. Ionic strength of the solution also affects the swelling of the hydrogels (Khare & Peppas, 1995). At a pH below the pKa, since the hydrogel is in the collapsed state, the effect of ionic strength on swelling is minimal. As the ionic strength increasing, the degree of swelling decreases for anionic hydrogels at a pH higher than the pKa of the polymer network (Khare & Peppas, 1995). Increasing the ionic strength of the solution results in ion shielding that diminishes the degree of electrostatic repulsion of the negative carboxylic acid groups (Shi et al., 2004).

Opposite to anionic hydrogels, cationic hydrogels are ionized at a pH below the pKa of the polymer network (Tahara et al., 2015). Cationic hydrogels are suited for drugs that release in the stomach or intracellular environments. Amino acid groups of cationic polymers impart high water solubility at acidic pH and low water solubility at neutral pH. Drugs are protected by cationic polymers in the oral cavity (pH 5.8–7.4), while releasing in the stomach (pH 1–3.5) (Yoshida et al., 2013) in an oral delivery system. Owing to the low solubility at neutral pH, suppressing drug release, cationic polymers often serve as taste-masking formulations (Douroumis, 2011, Yoshida et al., 2013). Example systems are summarized in Table 1.

**Table 1.** Categories of pH-responsive hydrogel with example polymers and applications for oral drug delivery.

	Polymers	Polymer type	Delivery site	Model drug and ref.
Anionic	P(MAA-g-EG)	Synthetic	Small intestine	Insulin (Bell & Peppas, 1996; Lowman et al., 1999; Ichikawa & Peppas, 2003), calcitonin (Torres-Lugo et al., 2002; Kamei et al., 2009), IFN-α (Kamei et al., 2009)
	P(IA-co-NVP)	Synthetic	Small intestine	Salmon calcitonin, urokinase, rituximab (Koetting et al., 2016)
	P(MAA-co-NVP)	Synthetic	Small intestine	siRNA (Knipe et al., 2016)
	Alginate-based	Natural	Small intestine and colon	Heparin (Huang et al., 2000), hemoglobin (George & Abraham, 2006), melatonin (Chen et al., 2004a), vaccines (Chen et al., 2004b; Kulkarni et al., 2001), peptides (Edelman et al., 2000), probiotic yeast (Rasmussen et al., 2003), cedroxil (Peppas & Huang, 2004)
	Hyaluronic acid-based	Natural	Small intestine	Insulin (Hurteaux et al., 2005), thrombin (Kim et al., 2002), α-chymotrypsin (Fiorica et al., 2013)
Cationic	Chitosan-based	Natural	Small intestine	Insulin (Li et al., 2016), BSA (Patel & Amiji, 1996; Kamei et al., 2009)
Amphiphilic	P(MAA-g-EG) with PMMA nanoparticles	Synthetic	Colon	Doxorubicin (Schoener et al., 2013)
Degrading polymers	Dextran-based	Natural	Colon	Hydrocortisone (Lee et al., 2008b), salmon calcitonin (Zhou et al., 2013)
	Gelatin-based	Natural	Small intestine and colon	5-fluorouracil (Anirudhan & Mohan, 2014)
	Carboxymethyl cellulose/poly(acrylic acid) hybrid hydrogels	Synthetic	Small intestine	Insulin (Gao et al., 2014)
	Maleic acid cross linked poly (vinyl alcohol)	Synthetic	Colon	Vitamin B12, salicylic acid (Basak & Adhikari, 2009)
	Azoaromatic crosslinks	Synthetic	Colon	siRNA, DNA (Chang Kang & Bae, 2011; Thambi et al., 2011) camptothecin
	BC-g-P(AA)	Combination of synthetic and natural	Small intestine	Insulin (Ahmad et al., 2016)
	Guar gum-poly(acrylic acid)-(-cyclodextrin) (GG-PAA-CD)	Combination of synthetic and natural	Small intestine and colon	Dexamethasone (Das & Subuddhi, 2015)

#### Hydrogels based on synthetic materials

Among hydrogel-based delivery systems, carboxylic acid containing polymers like poly(acrylic acid) (PAA) and poly(methacylic acid) (PMAA) offer many advantageous features for oral drug delivery, including pH-responsiveness, enzyme inhibition, mucoadhesion and the ability to open epithelial tight junctions (TJs) (Gao et al., 2014). The pH-responsiveness of PMAA grafted with poly(ethylene glycol) (PEG) tethers, denoted as P(MAA-g-EG), was first studied by Klier et al. and they further investigated the polymer network for applications in oral drug delivery systems (Klier et al., 1990; Peppas & Klier, 1991). An evaluation of grafted PEG chain lengths determined that PEG chains with a molecular weight of 1000 exhibited the highest degree of complexation in low pH (Bell & Peppas, 1996). Equimolar amounts of carboxylic acid groups of MAA and etheric oxygen molecules of PEG lead to the largest amount of complexation. Adjusting the amount of carboxylic acid groups or other substituent groups tailors the hydrogel system for a specific pH value and, therefore, the site of drug release.

Another important feature that takes advantage of the pH-responsive behavior of the P(MAA-g-EG) system is release of PEG tethers. In the decomplexed state, the grafted PEG tethers are no longer hydrogen bonding with carboxylic acids of the PMAA backbone and act as mucoadhesive promoters on the surface of the polymer network. Tethered PEG chains interpenetrate the mucus layer of the small intestine, participating in physical entanglement and hydrogen bonding with the polysaccharide components (Peppas & Huang, 2004). Mucoadhesion increases the residence time of the carrier at the site of absorption, which promotes increased bioavailability (Huang et al., 2000). It is important to note that pH-responsive of P(MAA-g-EG) hydrogel systems is designed for targeted release of drugs in the upper small intestine (Sharpe et al., 2014), as well as triggering the PEG tethers to promote mucoadhesion at the target absorption site.

P(MAA-g-EG) hydrogel systems have been used for the oral delivery of proteins, including IFN-α (Kamei et al., 2009), calcitonin (Kamei et al., 2009) and insulin (Ichikawa & Peppas, 2003), while modifications are necessary for hydrophobic molecules, such as chemotherapeutics. Amphiphilic polymeric carriers have been developed for oral delivery of hydrophobic drugs, especially doxorubicin, for targeted release in colon. This system combines the pH-responsive behavior of anionic complexation hydrogels with hydrophobic poly(methyl methacrylate) (PMMA) nanoparticles (NPs) incorporated into P(MAA-g-EG) networks. Increased PMMA incorporation leads to increased loading levels of doxorubicin and extended release for improved delivery to the colon (Schoener et al., 2013).

However, delivering proteins with high isoelectric points (pI) was hampered by coulombic interactions between the cationic protein and anionic hydrogel (e.g. P(MAA-g-EG)) in the small intestine, leading to binding rather than release for absorption into the blood (Carr et al., 2010). pH-Responsive poly(itaconic acid-co-N-vinyl-2-pyrrolidone) (P(IA-co-NVP)) was considered to be a potential carrier for drug delivery due to their favorable equilibrium swelling behavior in acidic and neutral pH environments (Betancourt et al., 2010). The additional carboxylic acid residue in itaconic acid can yield superior capability of swelling and drug delivery that would contribute to delivering high pI proteins, such as salmon calcitonin (Koetting & Peppas, 2014).

#### Hydrogels based on natural materials

Natural polymers, such as alginate, hyaluronic acid (HA) and chitosan, are attractive matrices for oral drug delivery due to their biocompatibility, physiochemical properties, and mild gelation conditions (George & Abraham, 2006). As an anionic polymer, alginate shrinks in a low pH conditions to form an insoluble alginic acid skin, which can change into a soluble viscous layer when exposed to higher pH environment of the intestinal tract. Interpenetrating networks of alginate with gelatin and egg albumin crosslinked with glutaraldehyde showed prolonged control release of cedroxil in *in vitro* studies (Kulkarni et al., 2001), which have been promised for protein oral delivery (George & Abraham, 2006). Studies have also used alginate as coated beads, plain beads and microcapsules for entrapping various biological molecules, including heparin (Edelman et al., 2000), melatonin (Benes et al., 1997), hemoglobin (Rasmussen et al., 2003), vaccines (Kim et al., 2002) and probiotic yeast (George & Abraham, 2006). HA, an anionic glycosaminoglycan, is also commonly used in drug delivery formulations. The presence of one carboxylic group per repeat unit imparts a pH-responsiveness, which is enhanced in crosslinked hydrogel network (Fiorica et al., 2013). The pH-responsive behavior of photocrosslinked HA hydrogels for the release of thrombin was evaluated by Pitarresi et al. (2004). A novel HA pH-responsive derivative with increased carboxylic groups was developed to optimize the system for drug delivery to colon with pH-responsive release using α-chymotrypsin (Sharpe et al., 2014).

Another natural polymer extensively used for drug delivery systems is chitosan, which is a cationic polymer extracted from crustacean chitin. Chitosan is considered as an efficient and safe intestinal absorption enhancer of therapeutic macromolecules, because of its pH-responsive, inherent biocompatibility, mild gelation conditions, mucoadhesive feature and ability to modulate the integrity of epithelial tight junctions reversibly (Muzzarelli et al., 1988). Owing to the amino groups on polymer chain, chitosan is protonated and easily dissolves at low pH, while insoluble at high pH. Therefore, chitosan has been extensively studied as a delivery vehicle for drugs to the stomach, and suitable for oral drug delivery by modifications (George & Abraham, 2006).

Although chitosan can be covalent crosslinking with dialdehydes (e.g. glyoxal (Khalid et al., 2002) and glutaraldehyde (Yamada et al., 2000)), ionically crosslinked with tripolyphosphate (Sun et al., 2011), chemically and mechanically reinforces the matrix, covalently crosslinked chitosan hydrogels are more stable for intestinal protein delivery. Further chemical modifications, such as trimethylated chitosan, thiolated chitosan, N-(2-hydroxyl) proyl-3-trimethylammonium chitosan and carboxymethyl chitosan, have been studied for oral delivery of bovine serum albumin (BSA) (Xu et al., 2003; Chen et al., 2004a), salmon calcitonin (Guggi et al., 2003) and various peptides (Sandri et al., 2005). The polyelectrolyte complexes of chitosan-alginate lead to decreased porosity, which is typical of alginate-only systems, and thereby reduces drug leakage (George & Abraham, 2006). Such complexes have been researched as pH-responsive hydrogels for the oral delivery of peptides and proteins, for example, hemoglobin (You et al., 2015).

#### Hydrogels based on combination of synthetic and natural materials

As the chemical initiators and crosslinkers used to synthesize hydrogels may be toxic, hydrogel based on combination of synthetic and natural polymers can be utilized to minimize the degradation of the polymers to smaller fractions in body (Ding et al., 2012). Bacterial cellulose (BC), a biopolymer synthesized by bacteria, was used due to its high mechanical strength, good water absorbance, and biocompatibility. Moreover, BC has good protein loading capability. The bacterial cellulose-g-poly(acrylic acid) (BC-g-P(AA)) hydrogel disks showed pH-responsive release of BSA and the potential to protect the structural integrity of loaded proteins *in vitro* (Ahmad et al., 2014). Insulin loaded BC-g-P(AA) hydrogel microparticles showed pH-responsive *in vitro* release and exhibited better hypoglycemic effect comparing to insulin solution, with improving relative oral bioavailability of insulin up to 7.45-time (Ahmad et al., 2016).

Guar gum (GG) is a natural polysaccharide which remains undigested in stomach and small intestine and is degraded to monosaccharides by the vast anaerobic microflora of the colon (Sinha et al., 2004). GG has also been usually conjugated with other polymers for forming interpenetrating polymer networks (IPN) to overcome the inherent drawback of the high hydrophilic characteristics. Generation of an IPN renders tougher thermal and mechanical characteristics to the otherwise fragile hydrogels. GG based IPN hydrogels have developed to combine with pH-responsive polymers, such as PAA with pluronic (Lo et al., 2013), PEG (Gu et al., 2013) and poly(vinyl alcohol) (Kurkuri & Aminabhavi, 2004), and explored for their efficacy in target specific drug delivery, such as dexamethasone for Crohn’s disease, ulcerative colitis and IBD.

### Nanoparticles

NPs have been extensively studied for oral delivery. NPs can protect encapsulated drugs from the low pH environment, drug efflux pumps, and enzyme degradation due to their stability in the GI environment. Recently, through cellular targeting with surface-functionalized ligands, transepithelial transport, and greater gastric retention, pH-responsive mechanisms have been included in novel nanomedicines to improve systemic exposure. One widespread approach to realize organ-specific drug release is to prepare NPs that exhibit pH-responsive swelling. For instance, when using acrylic-based polymers (e.g. PMAA), NPs retain a hydrophobic, collapsed state in the stomach because of carboxyl protonation. After moving though gastric passage, increasing pH results in NPs swelling due to the ionization of carboxyl groups and hydrogen bond breakage (Colombo et al., 2009). These characteristics enable PMAA-PEG diblock copolymers to achieve swelling ratios (mass of swollen polymer/mass of dry polymer) of 40–90-fold basing on PEG graft length and copolymer composition (Peppas, 2004). In such insulin loaded NPs, about 90% of the insulin was released at pH 7.4 within 2 h in their swollen state, while only 10% of the insulin was released at pH 1.2 in their collapsed state.

Surface-functionalized for NPs with acid-stable targeting ligands, including vitamins (Verma et al., 2016), lectin (Akande et al., 2010), and small peptides, for differential retention and uptake along the GI tract have been researched. Additionally, novel peptides were selected using *in vivo* phage display to identify peptides for targeted NP delivery to the M cells and follicle-associated epithelium (FAE) of the intestinal tract (Higgins et al., 2004). For instance, Arg-Gly-Asp (RGD) peptides were used to target β_1_ integrins expressed on the apical side of M cells *in vitro* (Gullberg et al., 2006) and *in vivo* (Garinot et al., 2007). Example systems are summarized in Table 2.

**Table 2.** Categories of pH responsive nanoparticles with example materials and applications for oral drug delivery.

	Materials	Delivery site	Model drug and ref.
Polyanions	Eudragits-based	Colon	Budesonide (Makhlof et al., 2009), Sulfasalazine (Kankala et al., 2015), curcumin-celecoxib (Gugulothu et al., 2014)
		Small intestine	CGP 57813 (Leroux et al., 1995), CGP 70726 (De Jaeghere et al., 2000), RR01 (De Jaeghere et al., 2001), cyclosporine A (CyA) (Dai et al., 2004)
	HPMCP	Small intestine	Insulin (Cui et al., 2007)
Polycations	Chitosan-based	Small intestine	Insulin (Rekha & Sharma, 2009, 2015; Cui et al., 2009)
The mixture of polyanions and polycations	Chitosan + Eudragit	Small intestine, colon	Insulin (Li et al., 2006, 2007; Jelvehgari et al., 2010; Chen et al., 2016), DNA (Momenzadeh et al., 2015), Psoralidin (Yin et al., 2016), Fluconazole (Rencber et al., 2016), CyA (Dai et al., 2015)
	Chitosan + poly(g-glutamic acid)	Small intestine	Insulin (Sonaje et al., 2010c), Amoxicillin (Chang et al., 2010)
	Chitosan + alginate	Small intestine	Bovine serum albumin (Chen et al., 2004b), Insulin (Mukhopadhyay et al., 2015)
	Chitosan + polyaspartic acid	Small intestine	5-fluorouracil (Zheng et al., 2007)
	Chitosan + poly (L-glutamic acid)	Small intestine	Doxorubicin (Deng et al., 2015)
	Chitosan + HPMCP	*in vitro*	Hepatitis B surface antigen(HBsAg) (Farhadian et al., 2015), Low-molecular weight heparin (Fan et al., 2016)
Inorganic materials	Nano-PSi + chitosan	Small intestine	GLP-1 co-loaded DPP4 inhibitor [No1]
	Nano-PSi + Eudragit	Small intestine	Fenofibrate (Jia et al., 2011), sorafenib (Wang et al., 2011), GLP-1 (Qu et al., 2012), Griseofulvin (Roine et al., 2015)
	Mesoporous silica nanoparticles (MSN)-based	Small intestine	Sulfasalazine (Lee et al., 2008a), Insulin (Guha et al., 2016)
	Calcium phosphate + chitosan + sodium alginate	Small intestine	Insulin (Verma et al., 2016)
Others	Polyacrylamide-grafted-xanthan gum (PAAm-g-XG)	Colon	Curcumin (Mutalik et al., 2016)

#### Nanoparticles based on polyanions

Eudragits, that is poly(methacrylic acid-co-methyl acrylate) copolymers, are widely used for pH-responsive NPs formulation. There are several types of Eudragits. Eudragit E100 is a cationic copolymer which dissolves in stomach, while Eudragit L100 and Eudragit S100 are anionic copolymers, separately dissolve at pH > 5.5 and pH > 7.0, therefore, they are applicable to ileal and duodenal drug release, respectively (Dai et al., 2004). Eudragit L100-55 containing an anionic copolymer dissolves at pH above 5.5 (Wang & Zhang, 2012).

In order to precisely control the drug release, multiple layers of pH-responsive Eudragits copolymers were used to coat over layered double hydroxide (LDH) NPs (Kankala et al., 2015). The LDHs used to immobilize drug molecules accelerate the dissolution of hydrophobic drugs significantly owing to increasing the drug surface area via highly dispersed drug molecules and decreasing the thickness of the diffusible layer via monolayer adsorption of the drug molecules in the LDH interlayers (Perioli & Pagano, 2012). Kankala et al. confirmed the effective intercalation of sulfasalazine, which is an anionic hydrophobic prodrug, into the interlayer of a LDH coating with pH-responsive Eudragit copolymer and with a high surface area leading to a typical specific and controlled release in the colon for the treatment of paw edema inflammation (Kankala et al., 2015). In addition, Eudragits mixed with some other polymers are usually used for NPs preparation.

Another anions polymers are also usually used as an enteric-coating agent, including HA and hydroxypropyl methylcellulose phthalate (HPMCP), such as HP50 and HP55, separately dissolve at pH 5.0 and 5.5. Recent research found pH-responsive HA NPs as a viable option for oral insulin delivery systems, showing enhanced delivery via transcellular pathway found in both *in vitro* and *in vivo* studies (Han et al., 2012).

#### Nanoparticles based on polycations

Similar to hydrogel, the cationic polymer used for preparing pH-responsive NPs is primarily chitosan, which can increase the absorption of NPs by the intestinal epithelium. As the solubility of chitosan limits drugs delivery to the intestine, different derivatives of chitosan have been developed with favorable characteristics, such as improving functioning also in a higher pH. These modified chitosan include quaternized chitosan (Siew et al., 2012), thiolated chitosan (Rekha & Sharma, 2015), carboxylated chitosan (Cui et al., 2009), amphiphilic chitosan (Rekha & Sharma, 2009), chitosan derivatives bearing chelating agents (Mourya & Inamdar, 2008), and PEGylated chitosan (Prego et al., 2006).

#### Nanoparticles based on combination of polyanions and polycations

Taking advantages of both polyanions and polycations, NP systems composed of the positive-charged chitosan and a negative-charged polymer have been developed, such as chitosan mixing with Eudragit (Li et al., 2006; Li et al., 2007; Jelvehgari et al., 2010), alginate (Chen et al., 2004b), polyaspartic acid (Zheng et al., 2007), methacrylic acid (de Moura et al., 2008), and poly(g-glutamic acid) (Sonaje et al., 2010a, 2010b, 2010c; Chang et al., 2010). A proper range of pH is needed to form NPs with polyanions and polycations. Beyond this range, NPs might collapse and release drugs. For instance, the chitosan/poly(g-glutamic acid) system was ionized and formed polyelectrolyte complexes at pH 2.5–6.6, leading to form NPs (Sonaje et al., 2010b), while the NPs subsequently disintegrated beyond this range (e.g. pH 1.2 and 7.4). This was because the ionized carboxyl group on poly(g-glutamic acid) tended to protonate at lower pH, while the quaternized amine groups on chitosan became deprotonated at pH above 6.5.

On the other hand, it is not an absolute requirement for cross-linker and homogenizer as the NPs can be prepared from two oppositely charged polymers, which provides a mild procedure to prevent drug (e.g. protein) denaturation (Jelvehgari et al., 2010), and improves oral absorption, especially the absorption at specific region, such as the colon. Recently, layer by layer (LBL) coated NPs have attracted considerable attention, which are composed of oppositely charged polyelectrolyte (like chitosan, alginate, polyacrylic acid, polyallylamine HCl, etc.) deposited over a core (Verma et al., 2016). These LBL coated NPs system have especially shown significant impact on stability and oral bioavailability related to protein delivery in GI tract.

#### Nanoparticles based on inorganic materials

Inorganic pH-responsive NPs have been reported in an increasing number of literatures in recent years due to their advantages in terms of rich variety, biocompatibility, thermal stability, and easy control of size, structure and morphology. One of these NPs with high potential is porous silicon (PSi) NPs. Besides the above superior properties, PSi NPs have tailor-made particle, high surface-to-volume ratio, top-down production, and easy surface modification which broadens their applicability to a great extent either by chemical conjugation or physical adsorption (Shrestha et al., 2014). In addition, minimal harsh condition avoids drug degradation during the drug loading process, therefore, especially suitable for the oral delivery of biomacromolecules. A novel pH-responsive nano-in-nano mucoadhesive PSi-based multifunctional nanosystem for dual protein-drug oral delivery, glucagon-like peptide-1 (GLP-1) co-loaded dipeptidyl peptidase-4 (DPP4) inhibitor, was developed by Shrestha et al. (Shrestha et al., 2015). This PSi-based nanosystem, conjugated with chitosan and coated with hydroxypropyl methylcellulose acetate succinate (HPMCAS MF), which is an enteric polymer, enables to withstand the hostile gastric environment and exhibited delayed release of the encapsulated peptide with enhanced intestinal permeability.

For the system of Eudragit and medical-grade nanoporous silica (Sylysia 350), the process of drug release might be more complex than that in the Eudragit NPs with two steps: i) Eudragit dissolved, a small part of drug released; and ii) silica exposed, drug embedded in the nano-pores diffused out and released (Wang et al., 2011). Such nanomatrix was prepared by an absolutely simple process of rotary evaporation. In the study for Cyclosporine A (CyA), the nanomatrix consisted of CyA, Sylysia 350 and Eudragit® S100 (1/5/5, w/w/w%) not only improved the dissolution of CyA *in vitro* but also displayed excellent enteric behavior. The CyA was highly dispersed in the nanomatrix in an amorphous or molecular state and partly filled into the nanopores of Sylysia 350. The relative bioavailability of optimized nanomatrix was 90.8% compared with Neoral® (Dai et al., 2015). Drugs with poor solubility, such as sorafenib (Wang et al., 2011) and fenofibrate (Jia et al., 2011) were also incorporated into the system. Therefore, this system has been successfully shown as platforms for NPs.

### Microspheres

Microspheres, derived from natural or synthetic materials, have been commonly studied for oral delivery of a wide variety of therapeutics. For example, polymeric microspheres of such as poly(methacrylic-g-ethylene glycol), calcium alginate (CA)-carboxymethyl cellulose, alginate and hyaluronate, have been used to stabilize insulin, exenatide, 5-FU, *Ganoderma lucidum* spore, etc. To further realize selective drug release in GI tract, materials include Eudragit S100, alginate, and poly(γ-glutamic acid) as well as their copolymers were used (Zhang et al., 2015), which may be formed microparticles by emulsion methods, self-assembly or other advanced technologies.

An additional issue limiting the practical applications of microspheres is the relatively hydrophilic nature of most enteric coating materials with hydroxyl, carboxyl or other polar moieties, which frequently cause the microspheres to display low drug loading capacity for many hydrophobic drugs (Cheng et al., 2012). To circumvent these issues, a one-step route based on guest-molecule-directed assembly of a structurally simple polymer via host-guest interactions was reported by Zhou et al., in which carboxyl bearing compounds (CBCs) are guest molecules (paclitaxel and indomethacin), while poly(N-isopropylacrylamide) serves as a host (Zhou et al., 2016). Different from generally pH-responsive delivery systems, the pH-responsiveness of these microspheres is mainly dominated by CBC molecules instead of carrier materials. These assembled microspheres have been proven to selectively release drug under intestinal conditions, with desirable scalability as well as excellent reconstitution capability, and may considerably improve the oral bioavailability of loaded therapeutics.

### Mini-tablets

Mini-tablets are very small tablets with diameter equal to or smaller than 3 mm, which can be placed in sachets or filled into a capsule shell for easy administration. They are easy to manufacture and can be coated so as to delay the drug release due to excellent smooth surface area, thus, they are considered as good substitutes for granules and pellets, and a possible modality for delivering medicines to children (Aleksovski et al., 2015). The pH-responsive mini-tablet for oral administration was first reported by Hu et al. Mini-tablets coated by P-4135F, a pH-responsive polymer with a higher dissolution threshold pH of 7.2 than the conventional polymers (e.g. Eudragit S100 and L100), was suggested to be useful for the delivery of norfloxacine to the lower part of the small intestine, i.e. the ileum (Hu et al., 1999).

Recently, Hadi et al. (2014, 2015, 2016) developed a novel pH-responsive coated mini-tablet filled capsule of naproxen for ileocolonic targeted drug delivery. These optimized mini-tablets were prepared by the direct compression method and were then coated with a 1:2 ratio of Eudragit L100 and Eudragit S100, respectively, with 20% coating level. By determining the pharmacokinetic parameters and *in vitro–in vivo* correlation (i.e. *R*_2_ = 0.9901) of the formulation, this mini-tablets showed suitable for targeted ileocolonic drug delivery. In their further work, 15 matrix-mini-tablets of naproxen were filled into an empty HPMC capsule, which possess all the advantages of a single unit bigger tablet and avoid the problems such as danger of dose dumping and alteration in release profile of drug due to unit-to-unit variation. This formulation, with drug content percentage to be 99.24 ± 0.10%, was found to be stable as per the guidelines of International Conference on Harmonisation of Technical Requirements of Pharmaceuticals for Human Use. As for pediatric use, Lou et al. developed a mini-tablet of cogrinded prednisone-neusilin complex. Coating mini-tablet cores with pH-responsive Euragite® EPO (Evonik) disabled drug release in simulated saliva, enabled rapid drug release in simulated gastric fluid and increased drug stability (Lou et al., 2013).

### Others

Besides oral drug delivery systems mentioned above, there are other novel systems demonstrated great potential for applications in the field of oral drugs delivery. For instance, electrospun nanofiber is regarded as a promising new formulation to the targets where is related to the changes of pH values owing to its unique features including versatility of drug incorporation, high loading efficiency, high surface area-to-volume ratio, and flexibility in surface functionalities (Ignatious et al., 2010; Xie et al., 2012). Jiang et al. developed polydopamine-coated PCL nanofibers encapsulated doxorubicin which could kill more cells at low pH compared to that at high pH values (Jiang et al., 2014). However, few studies have examined the pH-responsive electrospun nanofibers which could be due to the difficulty of fabrication of such smart fibers using electrospinning technique.

A single-unit dosage form with rhythmic delivery of therapeutic pulses may be suitable for disorder that exhibits a circadian rhythmic pattern. Considering the physiological conditions of the GI tract, site specificity of pulse delivery can be achieved by appropriately integrating the functions of pH-responsive and bacteria-responsive into a single unit. Sharma et al. designed a single-unit tablet in capsule device contained aceclofenac for the treatment of late night pain and morning stiffness associated with rheumatoid arthritis. Eudragit S100 was used as coating polymer for hard gelatin capsule as it displays pH-responsive solubility. The system was conceptualized as a three-component design: i) a hard gelatin enteric-coated capsule (for carrying two pulses), ii) first-pulse granules (for rapid release in intestine), and iii) second-pulse matrix tablet (for slow release in colon). The rapid-release pulse was aimed at relieving late night pain whereas the slow-release matrix tablet was targeted for drug release in colon to relieve early morning stiffness (Sharma & Pathak, 2013).

**Figure 1.**
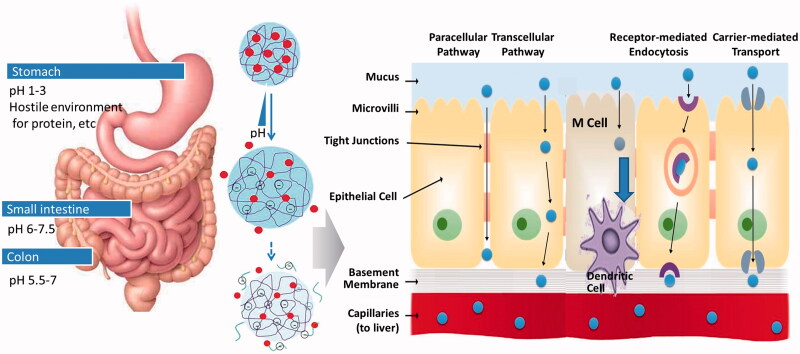
Drug release mechanisms and absorption process of pH-responsive oral delivery hydrogels/nanoparticles/microspheres (Wang & Zhang, 2012; Fox et al., 2015). Drugs release from pH-responsive hydrogels/nanoparticles/microspheres after the materials swelling and/or dissolution at specific pH. Drug molecules can cross the mucosal layer followed by a submucosal and areolar cell barrier where they interact with a plethora of transport pathways including paracellular or transcellular pathway or transcytosis pathway to enter systemic circulation. The paracellular pathway allows diffusion of molecules in the space between epithelial cells and is regulated by tight junctions formed between the cells. The transcellular pathway passes through the apical and basolateral cell membranes as well as the cytoplasm. It is restricted to hydrophobic molecules or molecules that have membrane pumps on the cell surface. The transcytosis pathway is an active transport pathway via receptor-mediated endocytosis and carrier-mediated transport. Transcytosis pathways are found in both epithelial and M cells. Particles on the scale of 1–1000 μm are not taken up by M cells (Kreuter, 1996), while particles of 50–1000 nm are phagocytized by M cells in Peyer’s patches. Only the size of the particles under 500 nm are used for cellular internalization in intestinal delivery to the systemic circulation (Moghimi et al., 2001; Sharpe et al., 2014), while particles <10 nm are cleared by lymph drainage (Moghimi et al., 2001).

In summary, various pH-responsive carriers have been developed for oral drug delivery. The release mechanisms and absorption process of these carriers were shown in Figure 1.

## Application of pH-responsive oral delivery systems

### Proteins and peptide therapeutics

Due to the complexity of macromolecules enables complex functions with a high degree of specificity unmatched by traditional small molecule drugs, proteins and peptide such as insulin, calcitonin and CyA, are experiencing the rapid increase in therapeutic application and result in more effective medicines with fewer off-target side effects. For example, the oral route replicates the pharmacodynamics of endogenous insulin release by entering the liver after intestinal absorption, similar to insulin secreted from the pancreas (Chen et al., 2013). The liver metabolizes 50–75% of insulin secreted from the pancreas, but only 25% of subcutaneous (SC) insulin (Arbit, 2004). The liver is more sensitive to insulin and acts faster in response to insulin to lower blood glucose levels; thus, less insulin is required to control blood glucose levels, even in diabetic patients (Quellhorst, 2002).

To date, it is encouraging to see that several oral protein and peptide loaded pH-responsive carriers have been produced by pharmaceutical companies and intended for application in clinical situations. Some of them have progressed to the clinical trial stage, such as Multi Matrix MMX® technology and CODES^™^ technology (Choonara et al., 2014). MMX® technology was produced for the oral delivery of active pharmaceutical agents (e.g. low molecular weight heparin) into the lumen of the colon by Cosmo Pharmaceuticals, Inc. (Lainate, Italy) and consists of tablets that are coated with pH-responsive acrylic copolymers which delay and control release. CODES^™^ technology was designed by Yamanouchi Pharmaceutical Co., Ltd. (Tokyo, Japan) for colonic specific delivery of insulin by lactulose-containing tablets coated with two acrylic films that exhibit pH-responsive solubility.

More researched are in the experimental stage by approaches such as receptor mediated endocytosis or mucoadhesion with pH responsive carriers, and most researches are focused on insulin and CyA. For improving the bioavailability of insulin, vitamin B12 (VitB12) conjugation with NPs has been used to further enhance the absorption of NPs by receptor mediated endocytosis in epithelial cells (Francis et al., 2005). These VitB12 conjugated NPs use body’s natural VitB12 transport system i.e. VitB12-IF-IFR (intrinsic factor receptor) which are present in ileocytes of intestine for systemic uptake of VitB12 (Petrus et al., 2009), and the pKa (∼1.8) of VitB12 leads change in zeta potential profiles of particles as function of pH. Verma et al. first reported the use of VitB12 in multilayered NPs. The results showed plasma insulin and blood glucose levels in diabetic rats were 4.3-fold increases in insulin bioavailability of administration with VitB12-chitosan-calcium phosphate NPs in comparison to chitosan-calcium phosphate NPs, and sustained hypoglycemic effects up to 12 h (Verma et al., 2016). In another study, chitosan, together with tripolyphosphate, poly(γ-glutamic acid), and MgSO_4_, was used to formulate “multi-ion-cross-linked” NPs. The NPs encapsulated insulin at pH <6 and released it at higher pH by chitosan deprotonization and NP destabilization. Multi-ion-crosslinked NPs had a superior stability over a broader pH range than NPs, and significantly more effectively transported insulin than NPs, suggesting that multi-ion-crosslinked NPs are a promising carrier for improved transmucosal delivery of insulin in the small intestine (Lin et al., 2008). pH-Responsive nanomatrix system of CyA with Sylysia 350 and Eudragit® attenuated the potential nephrotoxicity caused by the pronounced initial plasma peak of Neoral®, as well as enhanced the oral absorption of CyA and improved the relative bioavailabilities to 162.1% compared with Neoral, which could be attributed to fast stomach empting rate, absorption site specific, small degradation rate by luminal contents, high bioadhension of pH-responsive NPs to intestine mucosa and the use of P-Glycoprotein inhibitor if there is any (Wang et al., 2008). Examples of bioavailability improvement of insulin and CyA after orally administrating of different pH-responsive carrier are summarized in Table 3.

**Table 3.** Examples of relative bioavailability improvement of insulin and CyA after oral administration of different pH-responsive carrier.

Drugs	pH-responsive carriers	Relative bioavailability of insulin or CyA	Research object	Ref.
Insulin	PLGA-HP55 NPs	6.27% vs. SC injection	Diabetic rats	Cui et al. (2007)
	Chitosan NPs	14.9% vs. SC injection^a^	Diabetic rats	Pan et al. (2002)
	Chitosan and poly(g-glutamic acid) NPs	15.1% vs. SC injection	Diabetic rats	Sonaje et al. (2009)
	Chitosan and poly(g-glutamic acid) NPs filled in enteric-coated capsules	20.1% vs. SC injection	Diabetic rats	Sonaje et al. (2010a)
	[poly (methacrylic acid-co-vinyl triethoxylsilane)] coated mesoporous silica NPs	70.3%		Guha et al. (2016)
	Vitamin B12 functionalized layer by layer calcium phosphate NPs	26.9% vs. SC injection	Diabetic rats	Verma et al. (2016)
	Chitosan and poly(γ-glutamic acid) conjugated with ethylene glycol tetraacetic acid (γPGA-EGTA) NPs	17.8% vs. SC injection	Diabetic rats	Chuang et al. (2013)
	Bacterial cellulose-g-poly(acrylic acid) (BC-g-P(AA)) hydrogel microparticles	7.45-times vs. oral administration	Diabetic rats	Ahmad et al. (2016)
	Poly(ester amide) blend microspheres	5.9%	Healthy rats	He et al. (2013)
	Carboxymethyl cellulose/poly(acrylic acid) hydrogels	6.6% vs. SC injection	Healthy rabbits	Gao et al. (2014)
CyA	Nanoporous silica (Sylysia 350) and Eudragit® S100 nanomatrix	90.8% vs. Neoral	Rats	Dai et al. (2015)
	Eudragit S100 NPs	162.1% vs. Neoral	Rats	Yang et al. (2009)
	CyA-Eudragit® E100 NPs	94.8% vs. Neoral	Rats	Dai et al. (2004)
	CyA-Eudragit® L100-55 NPs	115.2% vs. Neoral	Rats	Dai et al. (2004)
	CyA-Eudragit® L100 NPs	113.6% vs. Neoral	Rats	Dai et al. (2004)
	CyA-Eudragit® S100 NPs	132.5% vs. Neoral	Rats	Dai et al. (2004)
	CyA-HP50 NPs	82.3% vs. Neoral	Rats	Wang et al. (2004)
	CyA-HP55 NPs	119.6% vs. Neoral	Rats	Wang et al. (2004)
	CyA-chitosan NPs	173% vs. Neoral	Beagle dogs	El-Shabouri (2002)

^a^Pharmacological bioavailability.

### Vaccination

Oral vaccines based on non-virulent peptides offer obvious advantages over parenteral injection routes. Although the absorption of orally delivered protein antigens through M cells in Peyer’s patches is very low caused by lack of specificity of antigens toward M cells and degradation of antigens in the GI tract, pH-responsive carriers is promising to be circumvented this limitation (Kim & Jang, 2014). For example, oral vaccines with an pH-responsive intelligent phase-transitional shielding layer, poly[(methyl methacrylate)-co-(methyl acrylate)-co-(methacrylic acid)]-PLGA (PMMMA-PLGA) was developed. During the protonation of weak basic radicals or ionization of weak acid radicals, pH-responsive swelling and changes in solubility occurred in polymers. The resultant PMMMA nano-shells, with pH regulated carboxyl responsive swelling and phase transition, may shield PLGA NPs from digestion in the stomach and small intestine, bypassing selective cellular uptake of the NPs in the small intestine, and then releasing PLGA/antigen NPs for cell uptake in the large intestine (Zhang et al., 2016). Mannan-modified pH-responsive poly(2-hydroxiethyl methacrylate-co-methacrylic acid) [P(HEMA-co-MAA)] nanogels (Duran-Lobato et al., 2014) and ileum-targeted delivery system using pH-responsive and mucoadhesive HPMCP were also synthesized and assessed as carriers for oral vaccines (Singh et al., 2015).

### Inflammatory bowel disease

IBD is a target for oral delivery. As opposed to most oral delivery applications that require the therapeutic to reach the bloodstream, the goal for IBD treatment is local delivery of therapeutics to immune cells in the intestines. Oral delivery strategies for IBD have attempted to take advantage of the pathophysiological processes associate with the disease to deliver therapies only at inflamed intestinal regions. So far, by encapsulating the drugs into pH-responsive oral formulations, many problems in treating IBD have been overcome, such as poor bioavailability, nonspecific tissue distribution, rapid elimination, poor retention in colon and related side effects.

Dew et al. developed the first colonic-targeted pH-responsive drug delivery system and it is most specifically referred to as “ileocolonic-targeted drug delivery” rather than a colonic targeted drug delivery system (Dew et al., 1982; Evans et al., 1988). Currently, colon-targeted systems are designed as multipleunit systems (mainly coated granules, pellets, microparticles and mini-tablets) for immediate or sustained drug release in this part of the GI tract (Srivastava et al., 2012). Our laboratory investigated a colon-specific capsule composed of Eudragit® RS PO, Eudragit® S100, GG and HPMC by a dipping process without coating. Radiolabeled with technetium-99m, this capsule remained intact in the stomach and small intestine and disintegrated in the proximal colon or the joint between the distal small intestine and right colon in volunteers. A large amount of radiolabeled marker was released and distributed in the whole colon after oral administration for 10 h (Han et al., 2009).

Another approach to target IBD is to create a synthetic polymeric vehicle responsive to both changes in pH and intestinal enzymes in inflamed intestinal regions. Knipe et al. create a synthetic polymeric vehicle responsive to both changes in pH and intestinal enzymes to impart targeted delivery of tumor necrosis factor-alpha small interfering RNA (TNF-α siRNA) to macrophages in inflamed intestinal regions (Knipe et al., 2016). These polycationic 2-(diethylamino)ethyl methacrylate (DEAEMA)-based nanogels were validated to facilitate cellular uptake and endosomal escape (Forbes & Peppas, 2014). The polycationic nanogels were encapsulated within an enzymatically degradable poly(methacrylic acid-coN-vinyl-2-pyrrolidone) [P(MAA-co-NVP)] hydrogel. The hydrogel should complex upon itself and protect the payload in gastric conditions but then swell, degrade, and release the nanogels complexed with siRNA in intestinal conditions (Knipe et al., 2015). After degradation, the size and surface properties of the nanogels are designed to facilitate accumulation in inflamed intestinal tissue where phagocytotic macrophages are present (Xiao & Merlin, 2012). These nanogels have been shown to facilitate endocytosis and subsequent endosomal escape of the siRNA payload, leading to siRNA delivery to the cytosol.

### Bacterial and viral infections

pH-Responsive drug delivery has been used to preferentially release drugs at sites of disease against bacterial and viral infections. For example, heparin-chitosan NPs were formulated to treat *Helicobacter pylori* infections (Lin et al., 2009). At pH 1.2–2.5, NPs were self-assembled by the mixing of chitosan and heparin, and remained stable in the gastric lumen owing to electrostatic interactions within the particles. Upon contact with an *H. pylori* infection along the gastric epithelium (pH ∼7.4), the deprotonation of chitosan led to weakened electrostatic interactions and resulted in NPs collapsing and heparin releasing.

## Conclusions and perspectives

pH-Responsive oral drug-delivery systems have been a research hotspot, and notable progress has been found over the past decades. Abundant experimental data has established a solid foundation but leave significant room for improvement, particularly in terms of increasing delivery specificity to the disease site and translation into clinical use. In order to implement the practice application, the ideal drug-delivery systems should have desirable multifunctionality to improve their performance in intelligent pH-responsive drug release, specific-site targeting ability, and diagnostic capabilities. Moreover, facile, low-cost, and controlled synthesis with well-defined structure, morphology, size, and chemical properties remains a great challenge. Novel pH-responsive oral drug-delivery systems by using biocompatible/biodegradable inorganic or inorganic/organic-composite nanostructured materials are crucial for practical use but have been relatively few reported.

Furthermore, inorganic materials, such as noble metals, metal oxides, rare earth oxides/fluorides, silica, and carbon (e.g. grapheme, carbon dots and carbon nanotubes), have displayed unique characteristics, such as high chemical/thermal stability, and have been investigated for biomedical applications. However, due to poor biodegradation behavior of these materials, their applications are limited *in vivo*. Accordingly, novel pH-responsive and biodegradable nanostructured inorganic materials with high biocompatibility, even nontoxic, are expected to provide promising applications for the oral administration, with the efforts to be made to improve the control of size, structure, morphology and drug loading. In addition, studies on evaluating the *in vivo* biotoxicity, biodegradability, and distribution pathways are necessary for their further applications.

Further researches are looking forward to exploring deeply for the processes in the physiological environment of pH-responsive carries both *in vitro* and *in vivo*, and more investigations should be conducted *in vivo* to further advance for their clinical applications. To study on the interactions between pH-responsive carriers and the drug molecules will be of great theoretical and practical significance for the design of novel oral drug delivery systems, with control over drug delivery, enhancement of drug loading capacity, and for further exploration of the possible mechanisms.
